# Mechanical, Computational, and Biological Evaluation of 3D-Printed PLA-Bioactive Glass Scaffolds for Bone Tissue Regeneration

**DOI:** 10.3390/ma19194080

**Published:** 2026-09-24

**Authors:** Andrea Martelli, Devis Bellucci, Andrea Nobili, Tian Li, Qaisar Nawaz, Aldo R. Boccaccini, Valeria Cannillo

**Affiliations:** 1 Department of Engineering “Enzo Ferrari”, University of Modena and Reggio Emilia, 41125 Modena, Italy; andrea.martelli@unimore.it (A.M.); andrea.nobili@unimore.it (A.N.); tian.li@unimore.it (T.L.); 2 Department of Materials Science and Engineering, Institute of Biomaterials, University of Erlangen-Nuremberg, Cauerstrasse 6, 91058 Erlangen, Germany; qaisar.nawaz@fau.de (Q.N.); aldo.boccaccini@fau.de (A.R.B.)

**Keywords:** bioactive glasses, composite scaffold, tissue engineering, cytocompatibility

## Abstract

Additive manufacturing enables the production of patient-specific biodegradable scaffolds for tissue regeneration. Polylactic acid (PLA), an FDA-approved polymer widely used in tissue engineering, suffers from hydrophobicity that limits cell adhesion, slows degradation, and may trigger inflammatory responses. To address these limitations, PLA was combined with bioactive glass (BG) particles to obtain printable composite filaments. Four BG compositions (45S5, S53P4, 45S5_MS, and S53P4_MS) were evaluated to determine whether Mg- and Sr-doped formulations improve biological performance of composites. The composites were characterized from morphological, physical, and mechanical standpoints, while scaffold wettability was further enhanced through alkaline surface treatment prior to in vitro cytocompatibility assays. BG incorporation increased brittleness and reduced compressive strength, while tensile properties remained comparable to pure PLA. Alkaline treatment effectively improved hydrophilicity and promoted cell adhesion and proliferation. Fluorescence microscopy revealed that scaffolds containing doped BG compositions supported particularly dense and well-spread cell populations. These findings demonstrate the synergistic effect of BG incorporation and surface modification in enhancing the performance of FDM-printed PLA scaffolds, supporting their potential use in bone tissue engineering.

## 1. Introduction

Nowadays, regenerative medicine (RM), the science concerned with restoring lost function, is becoming increasingly important in light of the global prevalence of age-related pathologies. In particular, tissue engineering (TE) represents the branch of RM that studies and designs methods for replacing biological tissues. In this context, scaffolds are 3D structures intended to support cell growth and proliferation during healing and, by virtue of their biodegradability, may progressively dissolve in the host tissue over time, thus eliminating the need of physical removal [1].

Additive manufacturing (AM) ranks among the most promising approaches to produce scaffolds, thanks to the high reliability of the samples obtained and the greater design freedom it offers [2,3]. Within AM, fused deposition modelling (FDM) has emerged as a fabrication technique for scaffold production. FDM enables solvent-free, high-throughput printing of complex, patient-specific architectures [4,5]. Several polymers can be used in FDM printing, and some of them are approved for in vivo use. Among them, polylactic acid (PLA) is a biocompatible, biodegradable, medical-grade thermoplastic polymer widely adopted in bone tissue engineering. Additionally, its relatively high elastic modulus and low elongation at break make PLA a good choice for bone substitution [5,6]. However, PLA exhibits some drawbacks. It has a relatively hydrophobic nature, resulting in water contact angles ranging from 75° to 85°, which may lead to poor cell affinity, slow degradation in aqueous media, and, at times, inflammatory responses in periprosthetic tissue [4,7,8,9,10,11,12,13]. In fact, cell adhesion and proliferation are influenced by the physical properties of the implanted material, such as surface wettability and bulk mechanical properties; by surface morphological characteristics, such as roughness and microporosity; and by chemical properties, including surface energy, surface charge, and surface-bound molecules [14].

To enhance the cell affinity of PLA surfaces, various treatments can be applied either to the bulk material—before or during polymerization, for example, through blending or physical/chemical modification—or to the surface, such as by coating, photo-grafting, or chemical modification [13,15], with the latter approaches directly modifying the final sample. An effective and simple way to modify PLA’s surface wettability and roughness is by alkaline hydrolysis [11,12]. Unlike other techniques, alkaline hydrolysis is a green, low-cost, and scalable process. This method involves the use of a strong alkali solution, such as NaOH or KOH, to degrade the PLA surface. This approach introduces hydrophilic functional groups (–COOH and –OH) on the PLA surface by cleaving the ester bonds of the aliphatic polyester [10,16]. Moreover, these functional groups can improve the attachment and proliferation of osteoblast-like cells [17,18,19].

To further tailor its mechano-biological response, PLA can be combined with a ceramic phase [4,7,20,21,22]. Among bioceramics, bioactive glasses (BGs) have attracted particular attention because their composition and amorphous nature enable them to readily interact with biological tissue, thereby promoting osteogenesis [23,24]. Notable examples include 45S5 Bioglass^®^ [24], the first BG discovered, and S53P4 Bonalive^®^ [25], both of which are commercially available and widely used in orthopaedic and dental applications. Furthermore, the physical and biological properties of BGs can be tuned by adding specific ions into their composition [26,27,28].

The combination of BG and PLA may help prevent inflammatory reactions while promoting a more favourable cellular environment by buffering the acidic degradation products of PLA with the basic degradation products of BG [29]. However, achieving a uniform dispersion of BG within the PLA matrix and obtaining a composite scaffold with BG exposed on the surface remains challenging when using a solvent-free approach [4]. Alkaline hydrolysis, acting on the surface, may facilitate the exposure of BG particles.

This work aims to investigate the combined use of PLA+BG composite scaffolds produced by FDM and alkaline hydrolysis to improve cell adhesion and proliferation. Specifically, four different BG compositions are considered, namely 45S5 and S53P4, alongside two novel BG materials derived from these commercial formulations, namely 45S5_MS and S53P4_MS. The latter were developed by incorporating Mg and Sr into the parent BG compositions. These novel BGs have previously been investigated in detail and demonstrated good in vitro cell viability and controlled conversion to hydroxyapatite [30]. In particular, the incorporation of Mg and Sr ions is expected to promote osteogenic activity and enhance processes associated with bone regeneration [31,32,33]. In the present study, these composite materials are analysed in terms of their physical and mechanical properties, while scaffold specimens are characterized both biologically and mechanically.

## 2. Materials and Methods

### 2.1. PLA+BG Composite Filament: Production

Composite filaments were manufactured by mixing PLA, serving as the bulk matrix material, with BG powders (5 wt%), serving as filler. PLA was purchased in granular form (PLA, 3Devo, Utrecht, The Netherlands). BGs were produced using a well-established melt-quenching method [30], as follows: all reagents (Carlo Erba Reagenti, Rodano-Milano, Italy) were accurately weighed and mixed for 2 h in a laboratory rotary mixer. The mixture was melted in a platinum crucible using the following thermal treatment: heating from room temperature to 1100 °C at 10 °C/min; holding at 1100 °C for 1.5 h for decarbonation; then heating to 1450 °C at 10 °C/min and holding at 1450 °C for 1 h to obtain a homogeneous melt. The molten glass was then quenched in water to achieve rapid cooling, resulting in the formation of a frit. The frit was dried at 110 °C for 12 h. Each frit was ground in a porcelain jar (PM100, Retsch, Haan, Germany), and the particle size was evaluated by laser scattering analysis (Zetasizer Nano ZS90—Malvern, UK). Glass compositions are detailed in Table 1.

In order to obtain a filament with constant diameter and homogeneously dispersed BG particles, a solvent-free method was used [4]. To prevent BG powder agglomeration, the BGs were sieved through a 63 μm mesh and treated with an antistatic ionizer (STABLO-AP, Shimadzu Corporation, Kyoto, Japan) before mixing with PLA. Then, the PLA–BG (5 wt%) mixtures were rotationally mixed at 60 rpm for 30 min. Additionally, to reduce the moisture content, the mixed raw materials were thermally treated at 60 °C for 24 h prior to extrusion.

The resulting composite mixture was introduced into the hopper of a desktop filament extruder (Filament Maker ONE, 3Devo, Utrecht, The Netherlands). To prevent heat-related material agglomeration, the material was fed in small portions, typically ranging from 10 to 20 g, thereby reducing the time spent in the hopper. This helped to maintain consistent material quality. During the extrusion process, the extrusion screw was consistently covered with layers of PLA+BG to ensure a steady intake of material. Cooling fans on the extruder were activated as soon as the filament reached a diameter of 1 mm. Once a stable target diameter was achieved, the filament was wound onto a spool. Filament diameters were then measured with a digital calliper, yielding a diameter of 1.75 ± 0.15 mm, considered suitable for subsequent scaffold printing.

Between each filament production cycle, the extruder was purged using high molecular weight polyethylene (HMWPE, 3Devo, Utrecht, The Netherlands) cleaning polymer to ensure that any residues from the previous run were removed. The extruder was equipped with four heaters whose working temperature, after some optimization, was set as follows: heaters 4 to 1 were maintained at 170, 190, 185, and 170 °C, respectively, with heater 4 positioned closest to the hopper and heater 1 nearest to the extrusion nozzle. The screw speed was set at 3.5 rpm, while the fan speed was adjusted to 70% of the extruder’s maximum fan speed. For comparison, a pure PLA filament was produced following the same protocol.

### 2.2. Materials Characterization

Filament morphology and BG particle distribution within the filament were evaluated by scanning electron microscopy (SEM) (Quanta 2000, FEI Co., Eindhoven, The Netherlands) on the gold-sputtered cross-section of the filaments (high vacuum, 25 kV, WD = 10.8 mm). To measure the actual volume fraction of BG within the composite filaments, the acquired images of the filament cross-sections were analysed with the public domain software ImageJ (v1.54g, NIH, Bethesda, MA, USA).

Additionally, the following formulas were used to calculate the theoretical volume fraction based on the composite constituents’ densities:

(1) $$\rho_{C}=\;\frac{1}{\frac{W_{m}}{\rho_{m}}+\;\frac{W_{f}}{\rho_{f}}\;}$$

(2) $$V_{f}=\frac{\rho_{c}}{\rho_{f}}\times W_{f}$$

where *ρ* represents the density, *W* denotes the mass fraction, and *V* corresponds to the volume fraction. In this context, the subscripts *c*, *m*, and *f* refer to the composite, matrix, and reinforcing phase, respectively. PLA true density was measured on PLA pellets prior to extrusion using a hydrostatic balance (E42S, Gibertini, Paderno Dugnano, Italy) with deionized water at 27 °C as the immersion medium. Conversely, the density of the BG powders was measured using a gas pycnometer (AccuPyc II 1340, Micromeritics Instrument Corp., Norcross, GA, USA). Helium with a purity of > 99.999 mol% was used as the analysis gas. For the calculation of composite density, an average value of BG density was considered.

To characterize extruded materials from a mechanical standpoint, dog-bone specimens were FDM-printed using the geometry shown in Figure 1. This specific geometry was selected to ensure a smooth transition from the gripping area to the testing area and thus promote failure in the mid-section. The geometry was designed using computer-aided design (CAD) software (SOLIDWORKS, v. 2023, Dassault Systèmes, Waltham, MA, USA) and sliced using a computer-aided manufacturing (CAM) software (PrusaSlicer 2.9.2, Prusa Research, Prague, Czech Republic).

Five dog-bone specimens of both PLA and PLA+BG, were printed using an FDM printer (Funmat Pro 310 Neo, Intamsys Technology, Ostfildern, Germany) equipped with a 400 μm diameter nozzle. The specimens were printed with 100% infill using an alternating ±45° raster pattern with respect to the tensile loading direction. The deposition direction was alternated between +45° and −45° in consecutive layers. The following printing parameters were used: base-plate temperature = 60 °C, printing temperature = 210 °C, and printing speed = 40 mm/s. To assess the presence of porosity within the dog-bone samples, the apparent density was measured using a hydrostatic balance with deionized water at 27 °C as the immersion medium. Then, the porosity was calculated using the following formula:

(3) $$Porosity=1-\;\frac{\rho_{app}}{\rho_{true}}$$

The hydrophilicity of PLA and PLA+BG samples was assessed using an Optical Contact Angle (OCA) system (Dataphysics, Filderstadt, Germany) with 2× magnification optics and 2 µL water droplets. Images and data were acquired via SCA20 software (v. 2.1.2, Dataphysics, Filderstadt, Germany). To modify surface hydrophilicity, a property closely linked to biological response, the samples were treated with 1 M NaOH for 30, 60, 90, and 120 min. Changes were evaluated by OCA, with ten measurements per sample.

Mechanical properties were tested under displacement control at 0.25 mm/min using a universal testing machine (UTM) (ProLine Z50, ZwickRoell, Ulm, Germany) equipped with a 10 kN load cell and wedge grips. Given the gauge length of 8 mm, traction occurs at 0.52 mstrain/s. A digital image correlation system (DIC) monitored the test using a digital camera (U3-31N0CP-M-GL, IDS, Obersulm, Germany) operating at 1 fps and equipped with a LED light source (Fiber Lite Mi-LED A2, Dolan-Jenner Industries, Boxborough, MA, USA): the test setup is shown in the Supplementary Materials (Figure S1). DIC frames were synchronized with the UTM displacement data, allowing each frame to be matched to a specific crosshead displacement and corresponding load cell force. By combining the DIC system with GOM Correlate (2018 edition), changes in the specimen strain field during loading up to failure were obtained. Images captured by the camera were imported into GOM Correlate and processed to determine the strain field at the corresponding time points.

Poisson’s ratio was evaluated according to the definition:

(4) $$\nu =\;-\frac{\varepsilon_{x}}{\varepsilon_{y}}$$

where εx and εy are the transverse and longitudinal strains, respectively. Poisson’s ratio was determined experimentally for each specimen at each DIC frame (corresponding to a specific time) within a reasonable time interval near, but not including, the origin—thus ensuring that linear deformations could still be assumed while excluding the initial setup adjustment phase [34].

Young’s modulus (E) was calculated from the stress–strain curve obtained from DIC analysis, which is far more representative of the real specimen response and gets away with the short flat (i.e., constant stress) stage that appears in the UTM stress–strain curve as a result of the gripping system adjustment. Stress was calculated from load data assuming a specimen cross-sectional area of 12.81 mm^2^. From Young’s modulus and Poisson’s ratio, the shear modulus (G) and the linear bulk modulus (K) were calculated according to the following equations, valid for isotropic materials:

(5) $$G=\;\frac{E}{2\left(1+\nu \right)}$$

(6) $$K=\frac{E}{3\left(1-2\nu \right)}$$

From the stress–strain curve, it is possible to deduce the non-linear moduli, in particular the neo-Hookean modulus. To this aim, a finite element (FE) analysis was carried out [34]. For a neo-Hookean material, the stored elastic energy is given by:

(7) $$W=\;C_{10}\left(I_{1}-3\right)+D_{1}{\left(J-1\right)}^{2}$$

where I_1_ = J^−2/3^tr(C) is the first deviatoric invariant of the Cauchy-Green deformation tensor C = F^T^F, and J = det F is the determinant of the deformation F. For consistency with linear elasticity, the following relationships must be satisfied:

(8) $$C_{10}=\;G/2,\;\;\;D_{1}=K/2$$

Thus, the model is fully defined from the linear moduli.

### 2.3. Scaffold Production and Characterization

The scaffold was designed using CAD software and printed by FDM. To promote effective cell infiltration, the scaffold had interconnected open porosity with a pore size of 600 µm, resulting in a surface area to volume ratio (SA:V) of 5.65 mm^−1^ [35,36].

The mechanical properties of the scaffolds were evaluated under compression with displacement control at 0.25 mm/min, corresponding to 0.42 mstrain/s, with a UTM equipped with a 10 kN load cell and compression plates. DIC system was also employed during the test.

Before biological evaluation, scaffolds were treated for 1 h in 1 M NaOH, as suggested by the contact angle results, to enhance cell attachment.

The in vitro cytocompatibility of the treated scaffolds was evaluated using a WST-8 cell viability assay. MC3T3-E1 pre-osteoblasts (passage 9, Sigma-Aldrich, St. Louis, MO, USA) were used as the model cell line. Prior to cell seeding, the scaffolds were sterilized under UV light for 1 h. Cells were cultured in α-MEM (Gibco, Waltham, MA, USA), supplemented with 10% fetal bovine serum (FBS; Sigma-Aldrich), 1% penicillin–streptomycin, and 1% glutamine. A total of 1 × 10^5^ cells were seeded directly onto each scaffold and incubated under physiological conditions (37 °C, 5% CO_2_) for 1 and 7 days. Cell viability was quantified using the WST-8 assay according to the manufacturer’s instructions. After each time point, cells were stained with Calcein-AM and DAPI (Life Technologies, Carlsbad, CA, USA), followed by fluorescence microscopy imaging (Axio Scope A.1, Carl Zeiss, Oberkochen, Germany) to evaluate cell attachment and morphology. All experiments were performed with n ≥ 3 samples and reported as mean ± standard deviation. Statistical differences between groups were assessed using a one-way ANOVA.

## 3. Results and Discussion

### 3.1. Materials Characterization

The densities of the bioactive glass powders were as follows: 45S5, 2.687 g/cm^3^; S53P4, 2.621 g/cm^3^; 45S5_MS, 3.015 g/cm^3^; S53P4_MS, 2.897 g/cm^3^. These results are consistent with those obtained for similar BG compositions [37,38,39]. An average BG powder density of 2.805 g/cm^3^ was considered, resulting in a composite density of 1.288 g/cm^3^. Based on these values, the theoretical volume fraction of BG was calculated to be 2.3%. In contrast, the volume fraction measured by image analysis of the filament cross-section was 1.72 ± 0.37%, corresponding to a relative error of 25.1%. This discrepancy between the theoretical and measured values may be attributed to the tendency of BG powder to form agglomerates, as previously reported and linked to residual surface charges [40,41], or to its adhesion to the extruder screw and barrel.

Density measurements on the dog-bone specimens revealed a discrepancy between the theoretical bulk material density and the actual specimen density. Specifically, the densities of the printed PLA and PLA+BG specimens were 2.4% and 7.2% lower than the corresponding theoretical values, respectively. This reduction in density can be attributed to the presence of internal pores introduced during the FDM printing process. Table 2 summarizes the density and porosity values measured for the dog-bone specimens.

Contact angle analysis, reported in Figure 2, highlighted a slight beneficial effect of BG addition to the PLA matrix in terms of hydrophilicity, consistent with the findings of Bernardo et al. [42]. This effect may result from surface modifications of the material and the formation of strongly polar areas due to the presence of BG particles on the surface [42,43]. Moreover, the results showed an effective reduction in the contact angle for PLA following alkaline treatment. This is in agreement with the literature and can be related to the presence of carboxyl and hydroxyl groups on the sample surface, known to improve wettability [10,11,44]. Interestingly, NaOH treatment of PLA+BG samples showed little to no effect up to 120 min. This behaviour may be explained by the presence of BG particles already exposed on the surface of untreated samples and by the progressive exposure of additional BG particles only after longer etching times, consistent with the non-linear relationship between etching and time [11]. Based on these findings, a 1 h treatment was deemed sufficient and selected to improve scaffold wettability.

The results of Poisson’s ratio evaluation for each DIC frame are reported in Figure 3, alongside the regression line. As expected, the measured Poisson’s ratio is almost independent of time, as the data points fluctuate only slightly and cluster around a stable mean, resulting in an almost flat regression line. Interestingly, for the representative specimens shown in Figure 3, Poisson’s ratio is 53% higher in PLA+BG specimens than in PLA specimens, increasing from 0.17 to 0.26. This is not surprising, considering the filling effect of the BG particles, which shifts the mechanical response towards the incompressibility limit, ν = 0.5. Notably, tuning Poisson’s ratio is particularly important for mimicking the mechanical behaviour of native tissue, as it affects the transverse deformation and, consequently, load transfer and local stress–strain environment experienced by cells and newly formed tissue [45,46].

Table 3 reports the mean values obtained for all specimens and the coefficient of variation (*CoV*) of the Poisson’s ratio and Young’s modulus for PLA and PLA+BG samples, together with the corresponding shear and bulk moduli. The results fall within the upper end of the range reported in the literature, which generally shows slightly lower values of Young’s modulus than those obtained here [47,48,49,50,51]. This discrepancy highlights the variability in mechanical properties introduced by the 3D printing process, mainly due to printing orientation or pore formation [47,48]. Accordingly, it is particularly important to experimentally determine material constants rather than relying solely on literature data.

Strength curves in terms of crosshead travel are plotted in the Supplementary Materials, Figure S3 for the PLA and PLA+BG groups, each comprising 5 specimens. The DIC analysis reveals that the crosshead travel is consistent with the imposed displacement rate, with the extension of the wedge grips taking place at the beginning of the test and accounting for the flat part of the strength curve in Figure S3. Through DIC, the crosshead displacement can be directly related to the effective strain in the specimen (specifically, in the mid-section), from which the nominal stress–strain curve can be obtained. This provides a more realistic representation of the material behaviour. Figure 4 compares the stress–strain curve of a representative PLA specimen and a PLA+BG specimen. In this representation, the plateau observed in the strength curve disappears, and the Young’s modulus can be readily obtained by taking the secant modulus of this curve.

The ultimate failure strain of PLA was experimentally measured at approximately 2.34%, although this value is highly dependent on the measurement position, the presence of defects, and the proximity to failure. Similarly, PLA+BG exhibited an ultimate failure strain of 2.30%. It is important to note that this is a pointwise assessment and does not correspond to the strain obtained by averaging over the gauge length, as used for the abscissa in Figure S3. Figure 5 shows the strain field just prior to failure, as acquired through the DIC system, with the coloured scale bar on the far right representing the colour legend for the DIC strain map. It indicates that both PLA and PLA+BG fail at nearly the same location near the mid-section, well outside the gripping area.

Table 4 summarizes the corresponding values derived from the strength curves and reveals that, as expected, the ultimate values generally underestimate the pointwise values obtained by DIC. Nevertheless, the greater brittleness of PLA+BG compared to PLA is confirmed by the lower UTE, while the ultimate force and UTS remain comparable between the two groups. Notably, the UTS values are consistent with those reported in the literature [48,49,50,52].

According to the FE model, as shown in Figure 6, the stress–strain response becomes a line that well represents the behaviour at small strain values but fails to capture the downturn observed in the experimental curve. Upon reaching the ultimate force of 600 N, the model correctly predicts a UTS of 43 MPa but underestimates the longitudinal displacement, predicting 0.48 mm instead of the expected 0.9 mm. Adopting the Mooney–Rivlin material does not provide much improvement.

### 3.2. Scaffold Characterization

Scaffold compression tests showed different failure modes for PLA and PLA+BG scaffolds. Figure 7a–d illustrates the progression to failure of PLA scaffolds, which fail by cell buckling along the weakest planes, leading to catastrophic instability. The overall process occurs in several steps, requiring sufficient damage accumulation to produce a pair of sliding planes. In contrast, PLA+BG scaffolds fail suddenly through catastrophic material failure, as shown in Figure 7e–g: the material reaches the critical strain simultaneously over a large volume, causing the specimen to fracture and resulting in an immediate loss of integrity. Overall, the results confirm that PLA+BG is considerably more brittle and weaker than PLA.

Table 5 reports the ultimate failure force (*F_u_*), stress (*σ_u_*) and strain (*ε_u_*) for PLA and PLA+BG samples. The more brittle response of the composite system compared to PLA is evident. PLA scaffolds consistently fail at a mean force approximately 7.4 times greater than that required to collapse PLA+BG scaffolds. Similarly, PLA fails at a strain level approximately 2.8 times higher than that of PLA+BG, with comparable data scattering. The latter, however, is significantly higher than that observed for force, as is typically the case for strain measurements. These findings are consistent with the literature [4,53]: the same strength vs. BG content trend was observed by Distler et al. [4], who reported lower compressive strength at higher BG content. These results suggest that, at this BG content, the particles did not reinforce the PLA matrix but may instead act as stress-concentration points.

However, the specific failure mechanisms cannot be conclusively identified based on the present analysis. Future studies will therefore include detailed microscopic analysis of the fracture surfaces to investigate the microstructural features associated with the failure mechanisms of the PLA-BG samples.

The cytocompatibility of the PLA and PLA–bioactive glass composite scaffolds (45S5, S53P4, 45S5_MS, and S53P4_MS) was evaluated using a WST-8 assay and fluorescence microscopy. Figure 8 shows the cell viability, expressed as a percentage, of MC3T3-E1 cells on the scaffolds after 1 and 7 days of incubation in cell culture medium. As shown in the viability graph, all scaffold types supported cell survival at both time points, confirming that neither the PLA matrix nor the incorporated BG particles induced cytotoxic effects [4,20,54]. After 1 day, cell viability levels were comparable across all groups, suggesting that the surface-treated scaffolds provided sufficiently favourable initial adhesion conditions regardless of the glass type. However, after 7 days, cell viability slightly decreased, although it remained within an acceptable range (>60%). Among the BG-containing scaffolds, those incorporating S53P4 displayed slightly higher metabolic activity than 45S5-based composites, which is consistent with the known ion-release behaviour of S53P4 and its ability to create a moderately alkaline microenvironment beneficial for osteoblastic activity [55]. This trend agrees with the material characteristics described above, where surface-exposed BG particles and NaOH-induced hydrolysis improved hydrophilicity and introduced –COOH/–OH groups that enhanced cell affinity of PLA-based scaffolds. Notably, all scaffold groups underwent the same alkaline surface treatment before cell seeding, which does not allow the independent contribution of this treatment to be quantified.

Fluorescence microscopy further supported the WST-8 results. Calcein-AM staining showed abundant viable cells adhering and spreading on all scaffold types, while DAPI revealed well-distributed nuclei across the filament surfaces and within the pore architecture (Figure 9). Cells displayed elongated morphologies, suggesting active cytoskeletal organization and stable attachment, particularly on BG-containing scaffolds, where the rougher, partially etched surfaces and exposed glass particles provided microscale anchoring sites. The modified-glass scaffolds (with 45S5_MS and S53P4_MS) exhibited qualitatively higher cell density and well-spread cells, which aligns with previous observations that Mg- and Sr-containing BGs promote osteoblastic proliferation through controlled ionic dissolution and enhanced surface reactivity [31,32,56]. Overall, the results confirm that surface-treated PLA and PLA+BG scaffolds are non-cytotoxic and promote early cell adhesion and proliferation, with S53P4- and S53P4_MS-containing scaffolds showing the most favourable biological response. However, quantitative cell adhesion measurements were not performed; therefore, differences in cell adhesion among the scaffold types cannot be statistically established. Further studies involving a quantitative assessment of cell adhesion will be necessary to determine whether BG composition influences this parameter. These findings highlight the synergistic effect of alkaline surface modification and bioactive glass incorporation on the biological performance of FDM-printed PLA scaffolds intended for bone tissue engineering applications.

## 4. Conclusions

In this work, the effect of incorporating BG particles into a PLA matrix for scaffold production was investigated. The mechanical properties of PLA and PLA+BG specimens were investigated both in tension using dog-bone specimens and in compression using scaffolds, exploiting a DIC system for accurate strain analysis. Tensile properties were little affected by BG incorporation, although the results suggest that BG particles may act as nucleation sites for crack initiation and propagation. Compared with the literature, the obtained results are generally higher—for example, in terms of stiffness—even for plain PLA. This may be due to the specific thermal history experienced by the material during FDM, as well as to the use of the DIC system, which, in combination with the UTM, provides a more accurate method for measuring mechanical properties than the commonly adopted approach, which solely relies on the crosshead travel for strain computation.

In contrast, the scaffold compressive properties of PLA+BG and PLA are markedly different: the presence of BG significantly affects ultimate strength, ultimate strain, and failure progression. PLA exhibits an ultimate compression strength approximately 7.4 times higher than PLA+BG, with an ultimate compression strain approximately 2.8 times greater, resulting in a markedly more ductile response. Furthermore, collapse in PLA+BG scaffolds occurs catastrophically when the material reaches its failure strain, causing the scaffold to fragment into small pieces—potentially beneficial for scaffold resorption or elimination. By contrast, PLA scaffolds fail through stability loss, preserving their overall integrity.

Overall, the biological evaluation confirmed that all surface-treated PLA and PLA–BG scaffolds were cytocompatible, supporting cell adhesion and proliferation without signs of cytotoxicity. Notably, the S53P4- and S53P4_MS-containing scaffolds elicited the most favourable cellular response, indicating their potential to enhance osteogenic performance in bone tissue engineering applications.

## Figures and Tables

**Figure 1:**
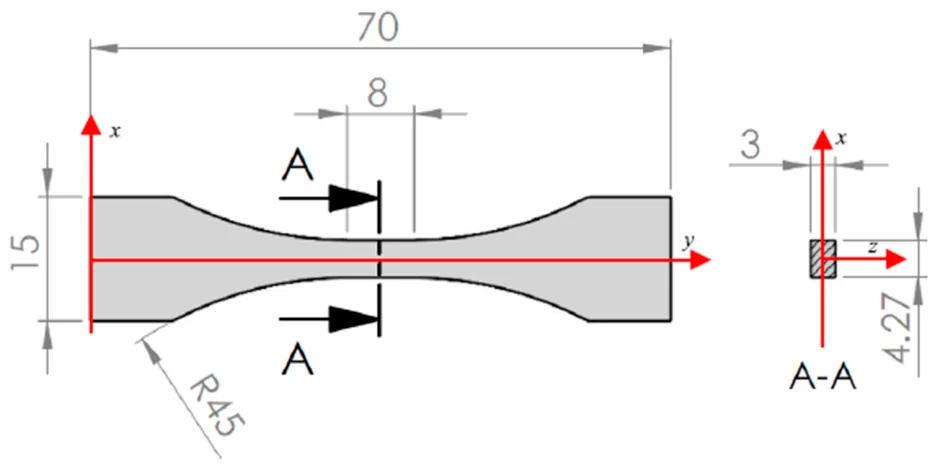
Dog-bone specimens for mechanical characterization in traction.

**Figure 2:**
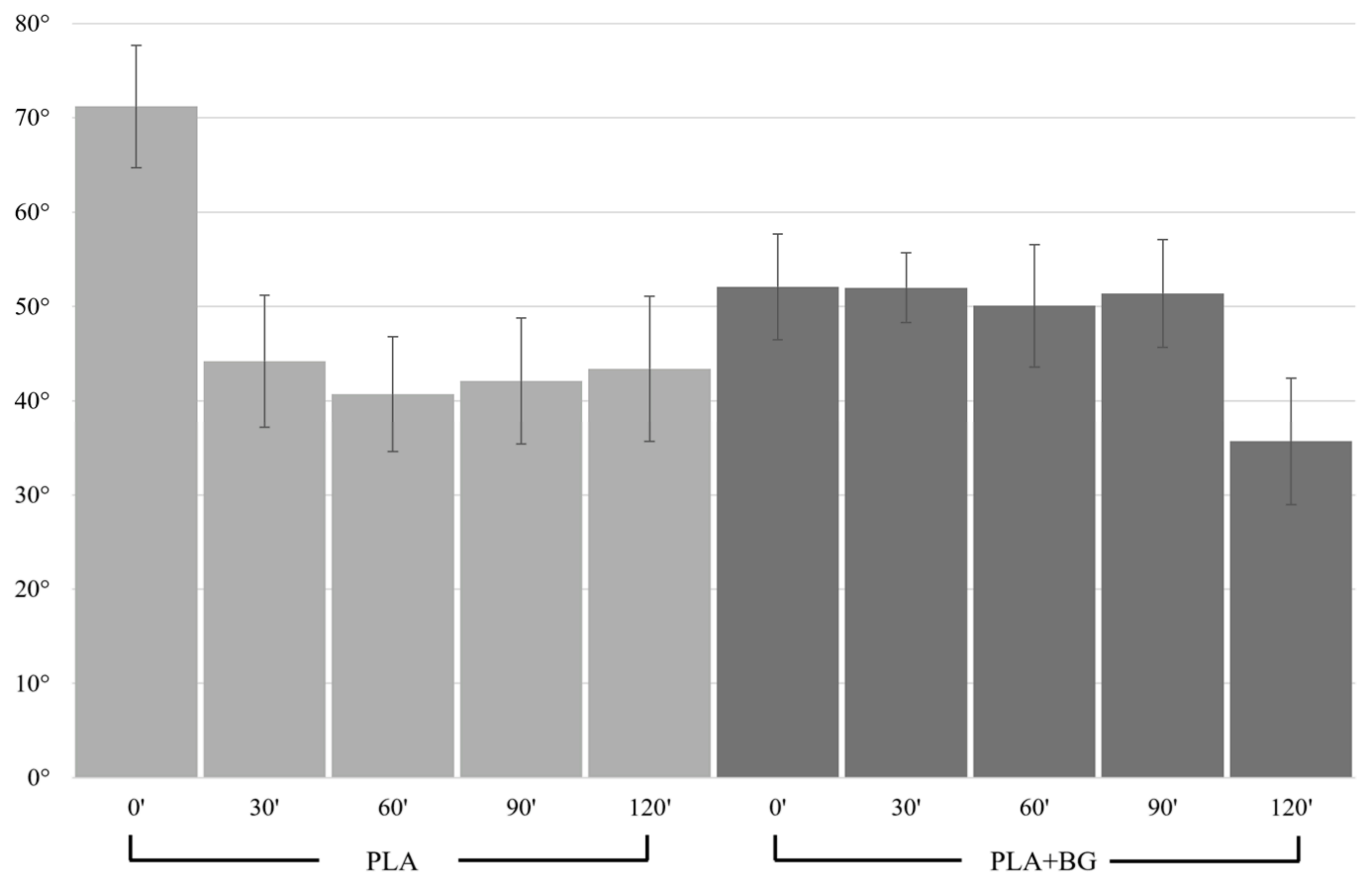
Contact angle measurements of PLA (light grey) and PLA+BG (dark grey) samples treated in NaOH 1 M for 0, 30, 60, 90, 120 min. The corresponding contact angle images are provided in the Supplementary Materials (Figure S2).

**Figure 3:**
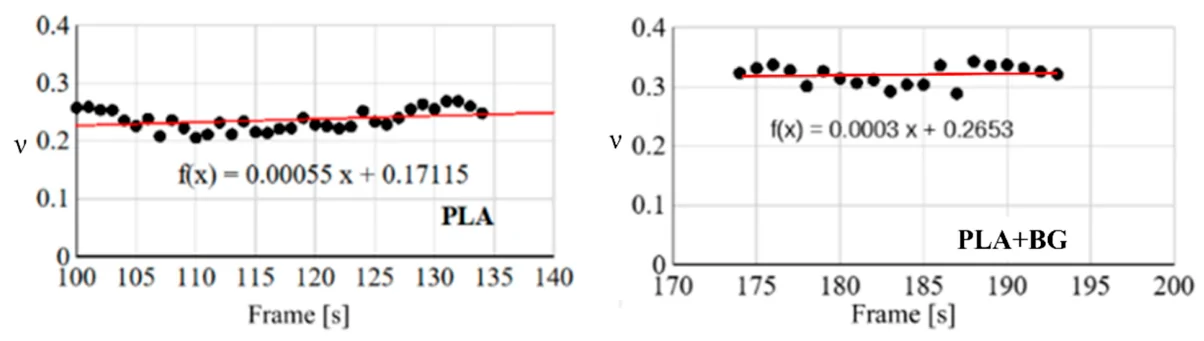
Poisson’s ratio for a typical PLA (**left**) and PLA+BG (**right**) specimen at different times (frames) and regression line which shows that the result is almost independent of time, at least for moderate strain.

**Figure 4:**
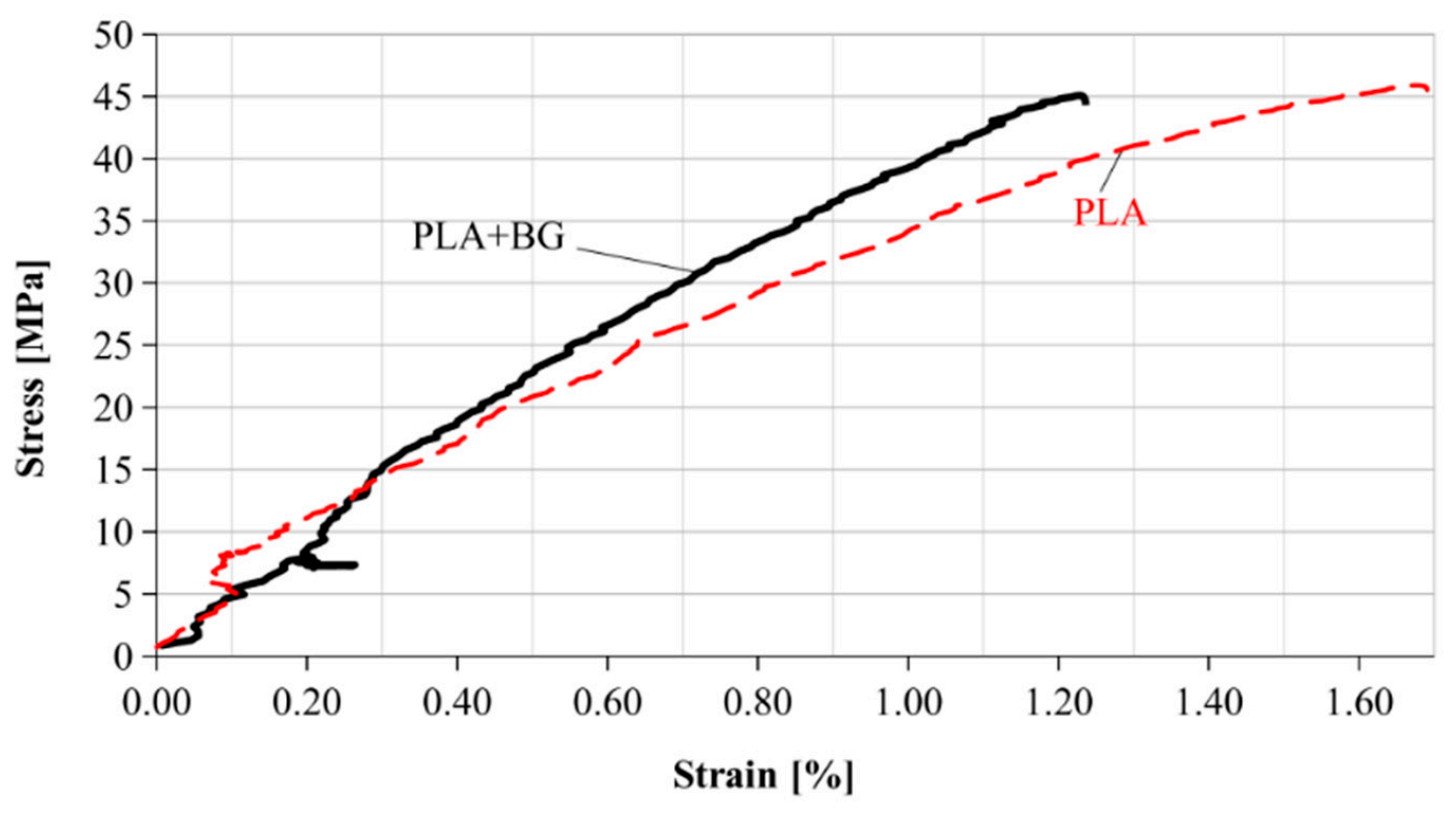
Stress–strain curve for PLA (red, dashed) and PLA+BG (black, solid) typical specimens. It is seen that the curves are similar and that the plateau, which is present in Figure S3 and reflects the action of the grips on the specimens, disappears because grip action does not affect the gauge material.

**Figure 5:**
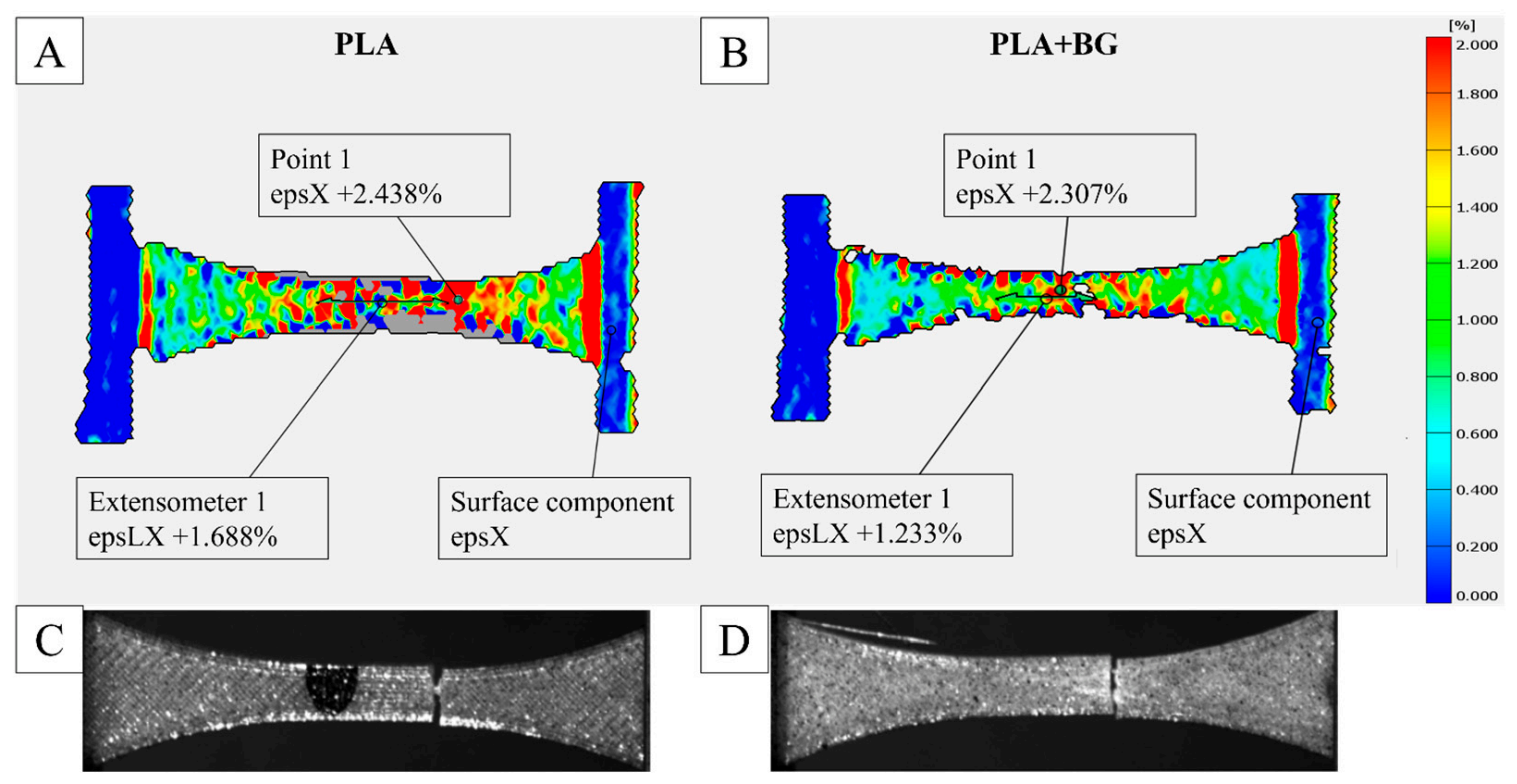
Strain field for a typical specimen of (**A**) PLA and (**B**) PLA+BG on the verge of failure. Failure in (**C**) PLA and in (**D**) PLA+BG occurs in almost the same location near mid-section.

**Figure 6:**
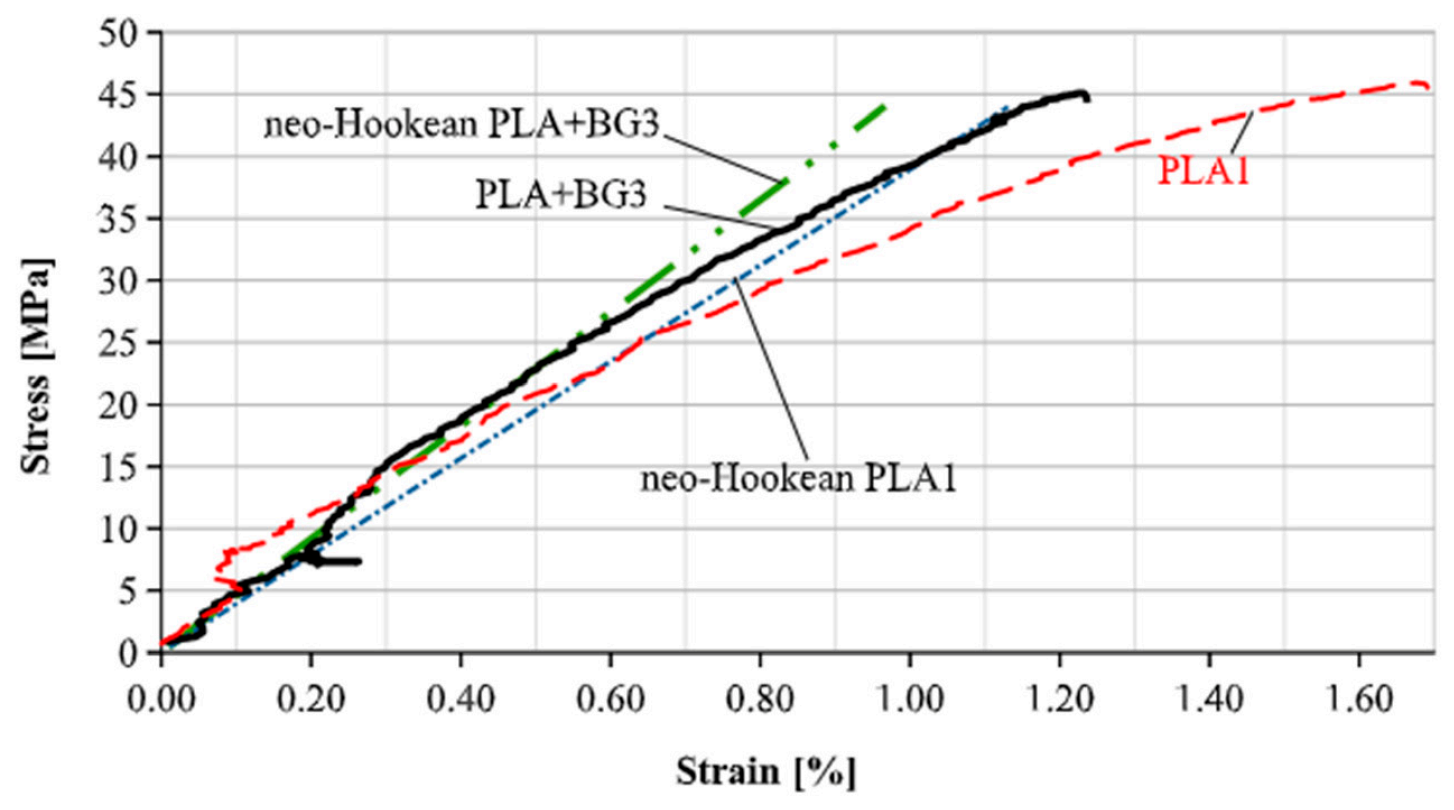
Stress–strain curve for PLA1 (red, dashed) and PLA+BG3 (black, solid) specimens and FE curve obtained with the neo-Hookean material model.

**Figure 7:**
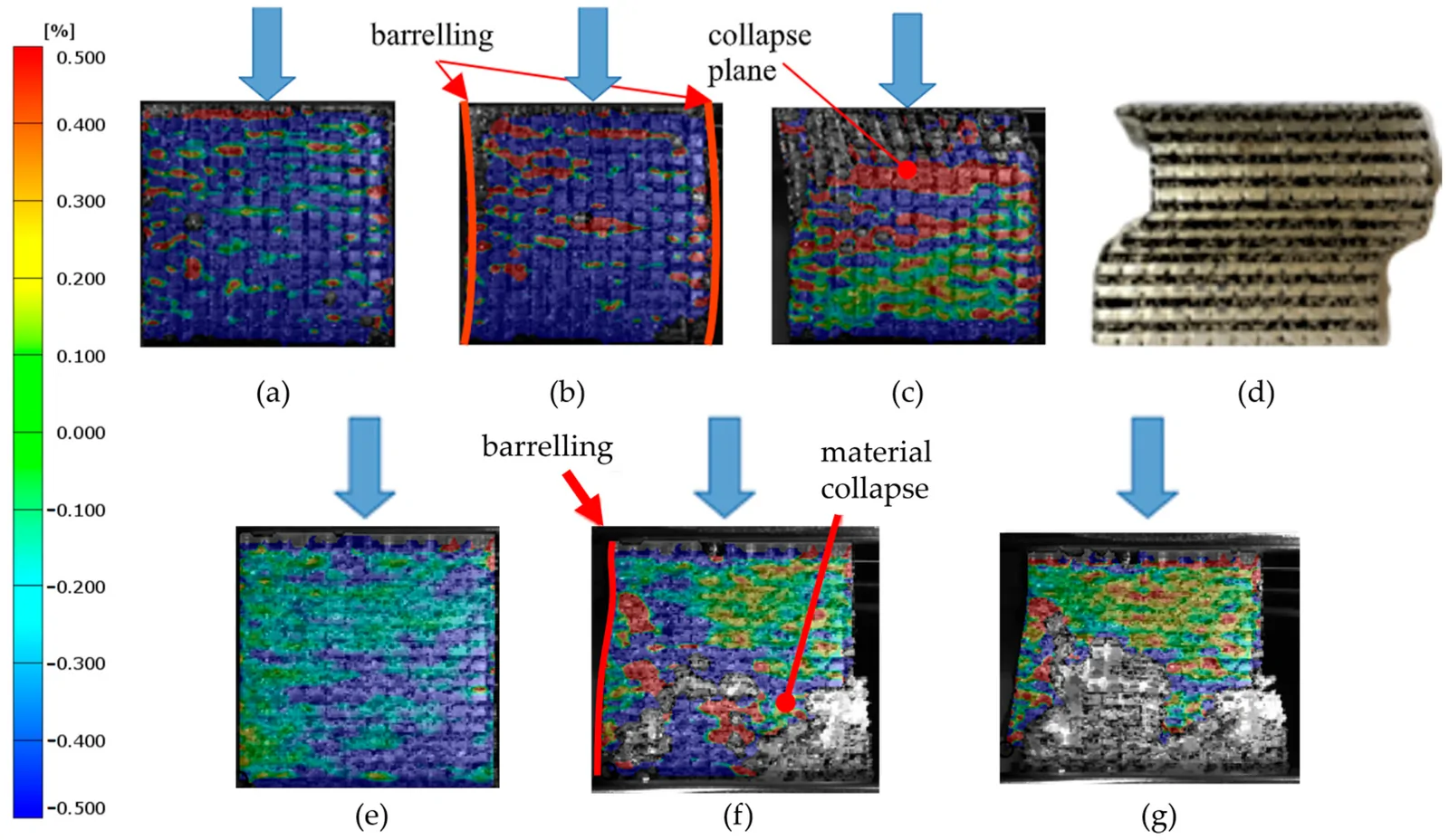
Scaffold progression to failure under compression, as acquired using the DIC system. The scale bar on the left represents the colour legend for the DIC strain map. PLA scaffold: (**a**) initial stage, with an almost homogeneous strain distribution, (**b**) specimen barrelling and strain localization, (**c**) strain concentration and consequent loss of stability, (**d**) collapsed shape. PLA+BG scaffold (**e**) initial stage, with a homogeneous strain distribution, (**f**,**g**) catastrophic material failure in the lower region of the scaffold, followed by overall collapse.

**Figure 8:**
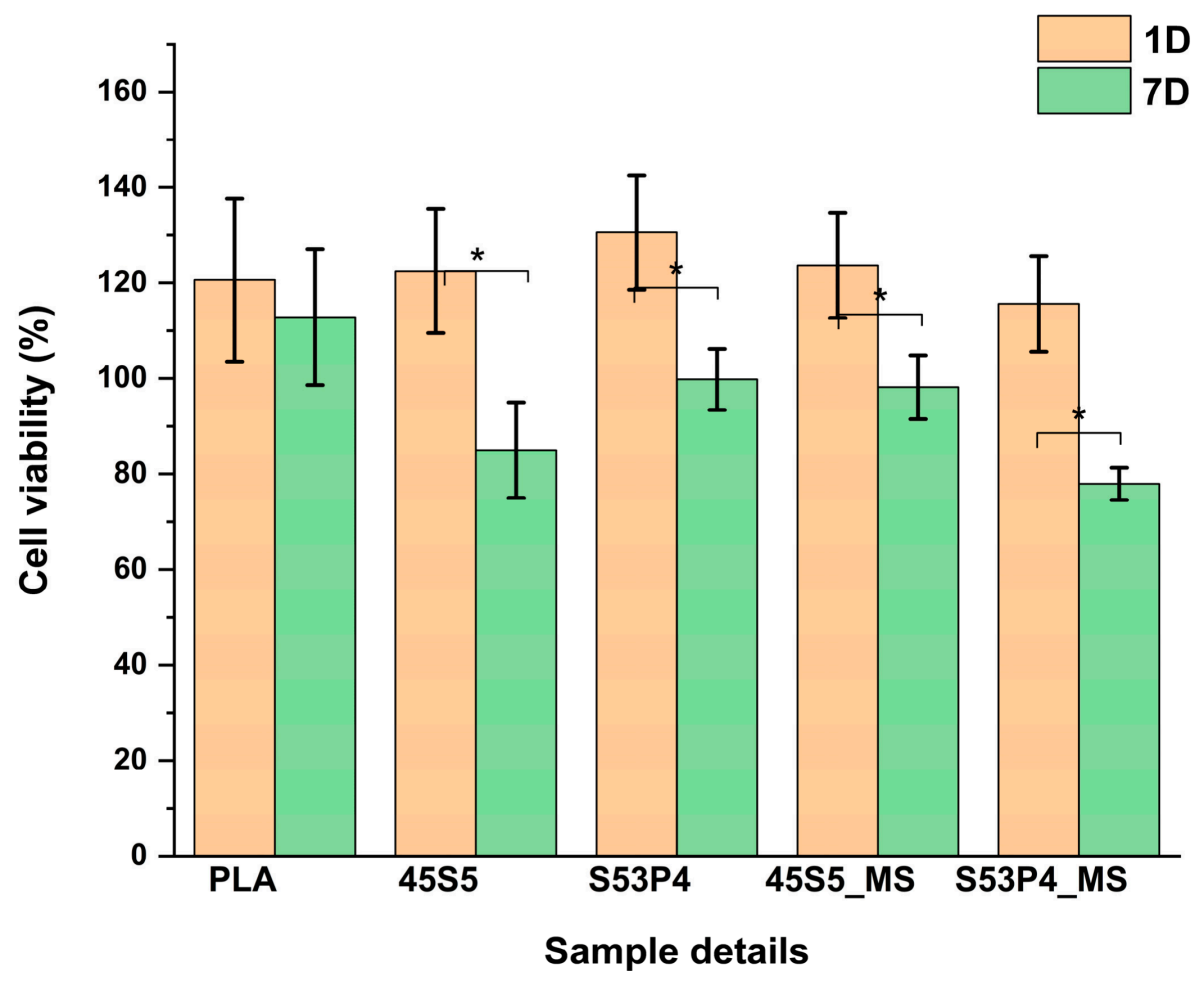
WST-8 cell viability at 1 and 7 days for scaffolds treated for 1 h in 1 M NaOH. Data are presented as mean ± standard deviation (SD). Statistical significance was determined using one-way ANOVA. Differences were considered statistically significant at *p* < 0.05. * denotes a statistically significant difference between Day 1 and Day 7 (*p* < 0.05).

**Figure 9:**
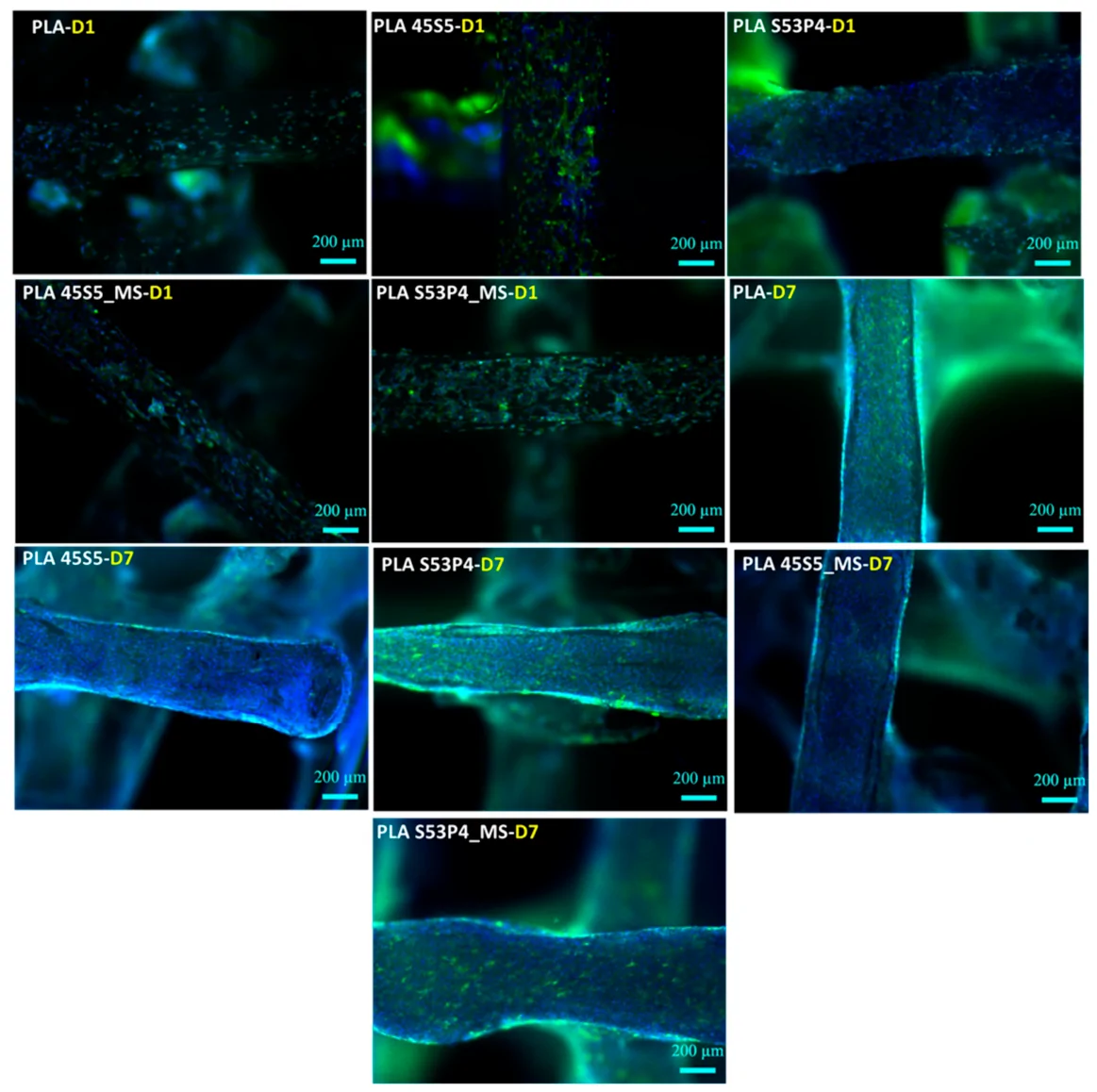
Fluorescence images of cells attached on scaffold surfaces after 1 and 7 days. Nuclei are shown in blue, and cytoskeletal or cellular structures in green.

**Table 1:** Bioactive glass compositions in mol%.

	Na_2_O	CaO	MgO	SrO	P_2_O_5_	SiO_2_
45S5 [24]	24.4	26.9			2.6	46.1
S53P4 [25]	22.7	21.8			1.7	53.8
45S5_MS [30]	9.4	26.9	5	10	2.6	46.1
S53P4_MS [30]	7.7	21.8	5	10	1.7	53.8

**Table 2:** Density and porosity values measured on dog-bone samples.

Sample	True Density (g/cm^3^)	Apparent Density (g/cm^3^)	Porosity (%)
PLA	1.2524 ± 0.0008	1.2226 ± 0.0006	2.4 ± 0.2
PLA+BG	1.2880 ± 0.0019	1.1958 ± 0.0011	7.2 ± 0.5

**Table 3:** Poisson’s ratio, ν, Young’s modulus, E, shear modulus, G, and bulk modulus, K, for PLA and PLA+BG specimens and their statistical significance.

Sample	ν	CoV (%)	E (MPa)	CoV (%)	G (MPa)	CoV (%)	K (MPa)	CoV (%)
PLA	0.232 ± 0.012	5.03	3934 ± 325	8.27	1596 ± 85	5.35	2446 ± 150	6.12
PLA+BG	0.322 ± 0.019	5.97	4591 ± 340	7.40	1736 ± 102	5.89	4299 ± 278	6.47

**Table 4:** Ultimate Tensile Elongation (UTE) and Stress (UTS) for PLA and PLA+BG together with mean, standard deviation and coefficient of variation (CoV).

Sample	UTE (%)	CoV (%)	Force (N)	CoV (%)	UTS (MPa)	CoV (%)
PLA	1.72 ± 0.20	11.62	565 ± 17	3.08	44.83 ± 1.37	3.05
PLA+BG	1.41 ± 0.11	7.79	575 ± 3.82	0.66	44.89 ± 0.30	0.66

**Table 5:** Failure compressive force, stress, strain for PLA and PLA+BG scaffolds.

Sample	F_u_ (N)	σ_u_ (MPa)	CoV (%)	ε_u_ (%)	CoV (%)
PLA	5169 ± 386	12.92 ± 0.97	7.47	10.21 ± 2.73	26.76
PLA+BG	699 ± 70	1.75 ± 0.18	9.98	3.60 ± 0.38	10.51

## Data Availability

The original contributions presented in this study are included in the article/Supplementary Material. Further inquiries can be directed to the corresponding authors.

## Supplementary Materials

The following supporting information can be downloaded at: https://www.mdpi.com/article/10.3390/ma19194080/s1, Figure S1: Tensile test setup: UTM with wedge grips, DIC camera and light source (left), specimen area (right); Figure S2: Contact angle measurement on PLA and PLA-BG samples treated in NaOH 1M for 0, 30, 60, 90, 120 min; Figure S3: Strength curves in terms of crosshead displacement (standard travel in mm) for PLA (left) and PLA+BG (right) specimens.
