# Supplementary material for: Mechanical, Computational, and Biological Evaluation of 3D-Printed PLA-Bioactive Glass Scaffolds for Bone Tissue Regeneration
Source: Materials (Basel). 2026 Sep 24;19(19):4080. doi: 10.3390/ma19194080 (PMC13665739; doi:10.3390/ma19194080)
Supplement: Supplementary file 1 [file materials-19-04080-s001.zip › materials-4494705-supplementary.pdf]

Supplementary material:

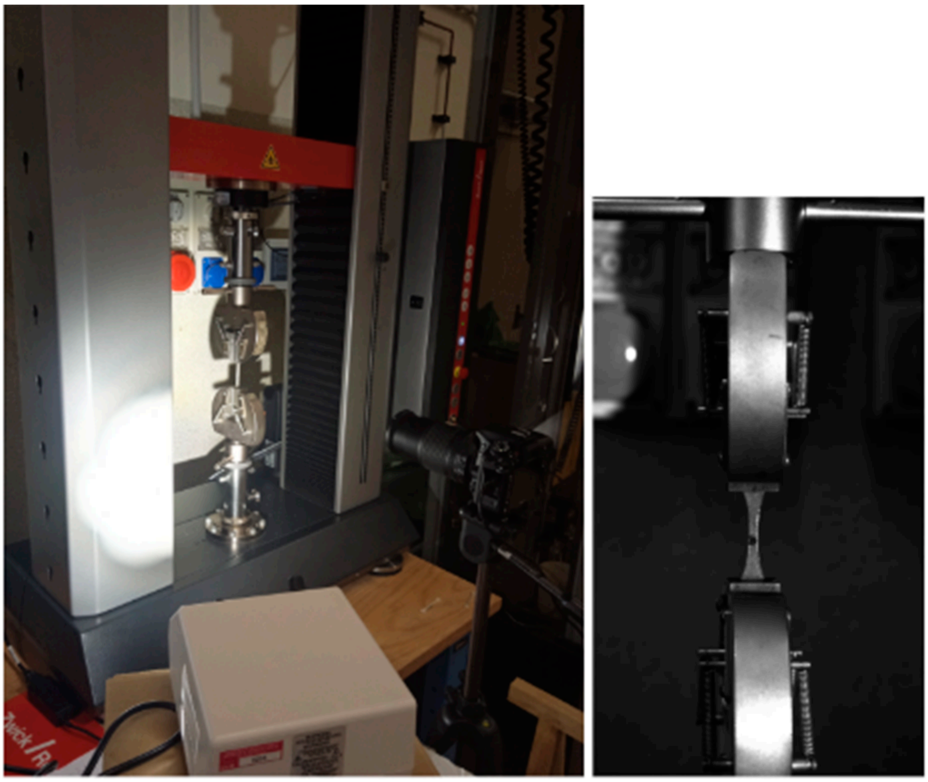

Figure S1 *Tensile test setup: UTM with wedge grips, DIC camera and light source (left), specimen area (right)*

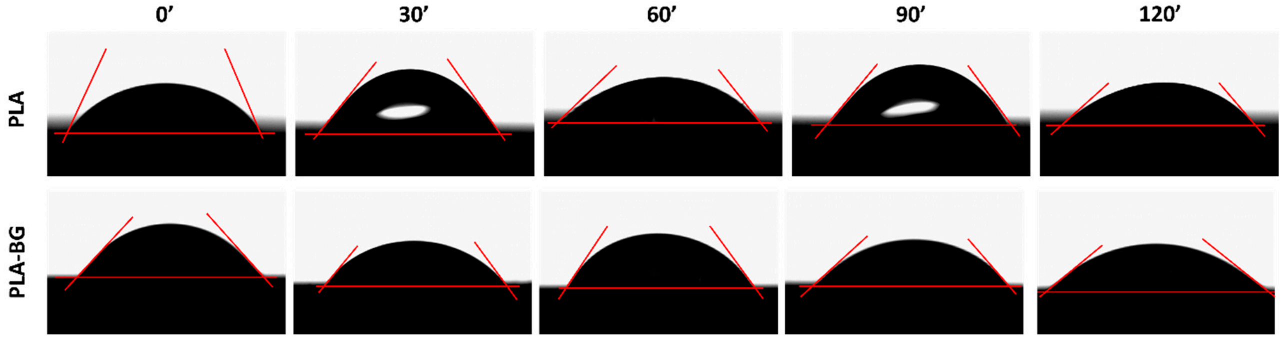

Figure S2 *Contact angle measurement on PLA and PLA-BG samples treated in NaOH 1M for 0, 30, 60, 90, 120 minutes.*

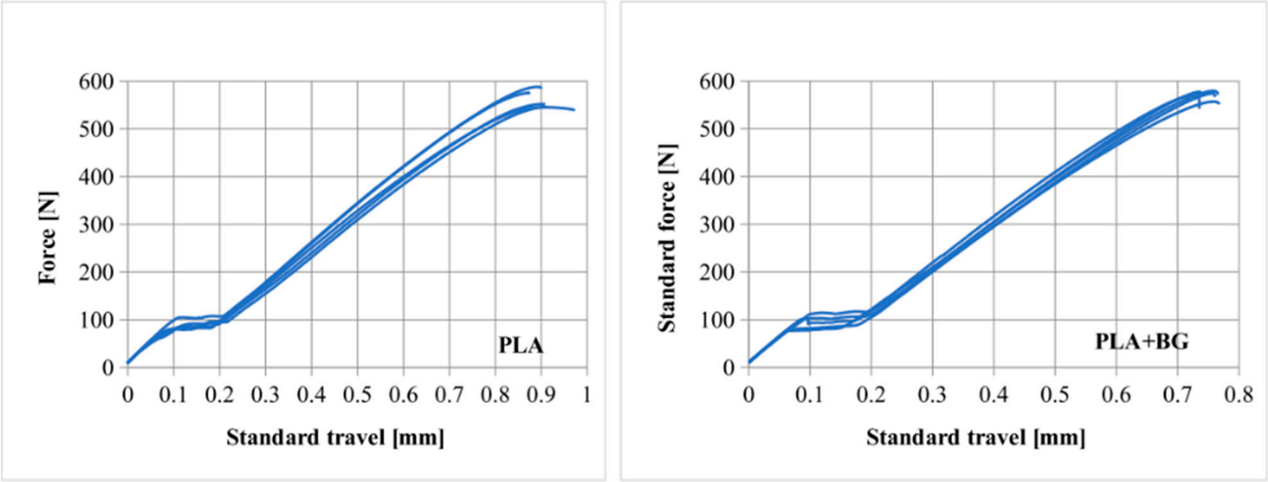

Figure S3 *Strength curves in terms of crosshead displacement (standard travel in mm) for PLA (left) and PLA+BG (right) specimens. Five repetitions are presented in each group which are well consistent. The plateau in the plots is a result of the compression that the grips produce on the specimen ends, that induces elongation at an almost constant force.*
